# An ex vivo Model of Paired Cultured Hippocampal Neurons for Bi-directionally Studying Synaptic Transmission and Plasticity

**DOI:** 10.21769/BioProtoc.4761

**Published:** 2023-07-20

**Authors:** Ruslan Stanika, Gerald J. Obermair

**Affiliations:** Division of Physiology, Department of Pharmacology, Physiology, and Microbiology, Karl Landsteiner University of Health Sciences, Krems, Austria

**Keywords:** Primary hippocampal culture, Paired patch clamp recording, Synaptic transmission, Calcium channel, α_2_δ subunit, Viral infection, Electrophysiology, Short-term plasticity, Postsynaptic currents, Paired pulse facilitation and depression

## Abstract

Synapses provide the main route of signal transduction within neuronal networks. Many factors regulate critical synaptic functions. These include presynaptic calcium channels, triggering neurotransmitter release, and postsynaptic ionotropic receptors, mediating excitatory and inhibitory postsynaptic potentials. The key features of synaptic transmission and plasticity can be studied in primary cultured hippocampal neurons. Here, we describe a protocol for the preparation and electrophysiological analysis of paired hippocampal neurons. This model system allows the selective genetic manipulation of one neuron in a simple neuronal network formed by only two hippocampal neurons. Bi-directionally analyzing synaptic transmission and short-term synaptic plasticity allows the analysis of both pre- and postsynaptic effects on synaptic transmission. For example, with one single paired network synaptic responses induced by both, a wild-type neuron and a genetically modified neuron can be directly compared. Ultimately, this protocol allows experimental modulation and hence investigation of synaptic mechanisms and thereby improves previously developed methods of studying synaptic transmission and plasticity in ex vivo cultured neurons.

Key features

Preparation of ex vivo paired cultured hippocampal neurons.

Bi-directional electrophysiological recordings of synaptic transmission and plasticity.

Genetic modulation of synaptic network formation (demonstrated by presynaptic viral overexpression of the auxiliary calcium channel α_2_δ-2 subunit).

Graphical overview

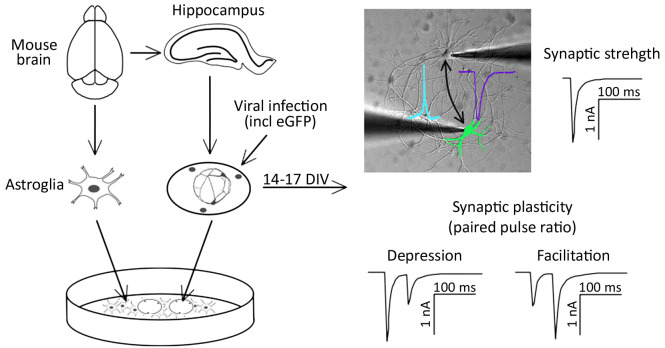

## Background

Signal transmission between neurons occurs via neurotransmitter release into the synaptic cleft. The temporal and spatial relation of pre- and post-synaptic firing modulates the strength of synaptic connections between neurons (Deperrois and Graupner, 2020). Two types of synaptic activity can be registered at the single cell level. First, miniature excitatory or inhibitory postsynaptic currents (mEPSC or mIPSC, respectively) appear as result of spontaneous local fusion of single synaptic vesicles. Second, excitatory and inhibitory postsynaptic currents (EPSC or IPSC, respectively) can be recorded in response to action potential firing by presynaptic glutamatergic or GABAergic neurons, respectively. Analysis of miniature postsynaptic potentials provides information on the amount and density of synaptic connections (frequency), as well as the postsynaptic receptor abundance (amplitude), and hence helps to study elementary synapse properties. However, higher levels of synaptic function, including the responses of synapses in regard to action potential firing, as well as short- and long-term adaptations of synaptic strength, require the analysis of evoked synaptic transmission. For example, paired-pulse stimulation protocols can serve as a basic model for studying short-term synaptic plasticity ex vivo (Bouteiller et al., 2010). Evoked synaptic transmission and plasticity in specific neuronal pathways can typically be studied in brain slices (Wang and Baudry, 2019). Alternatively, synaptic transmission and plasticity can be studied in cultured neurons, such as by employing optogenetic activation of neuronal cell populations (Barral and Reyes, 2017). In an acute brain slice, which is the standard model for the electrophysiological analysis of synaptic functions, presynaptic stimulation and postsynaptic responses can only be analyzed in one direction. However, as synaptic plasticity involves the possibility of changes in both pre- and post-synaptic components, one-directional measurements limit the study of mechanisms involved in modulating plasticity.

Here, we describe a protocol for culturing simple networks of paired hippocampal neurons for the bi-directional electrophysiological analysis of synaptic functions. This cellular ex vivo model has the following advantages: first, it allows the easy identification of the innervated cells. In classical neuronal cell cultures employing dispersed neurons, this is inherently difficult due to excessive branching of the axons and the possibility of hetero-synaptic innervation of the target neuron. Second, due to the defined simple network, all synapses are formed between the paired neurons. This results in increased amplitudes of postsynaptic responses and hence allows the reliable detection of changes in postsynaptic receptor function. Third, both cells of the cultured paired network can function as presynaptic (stimulated) and postsynaptic (innervated) neurons. Hence, this method allows recording synaptic transmission bi-directionally. This is particularly relevant in the context of genetic manipulations of one of the two paired neurons: as one of the paired neurons can be genetically modified by overexpression or knockdown of specific proteins, bi-directional stimulation protocols allow analyzing pre- and post-synaptic consequences in comparison with wild-type synaptic connections in the same neuronal network. As a proof of principle, we altered the expression of α_2_δ proteins, which act, on the one hand, as auxiliary subunits of voltage-gated calcium channels (Geisler et al., 2015; Ablinger et al., 2020; Dolphin and Obermair, 2022), and on the other hand as critical synaptic organizers (Eroglu et al., 2009; Geisler et al., 2019; Schöpf et al., 2021; Ablinger et al., 2022). Hence, we employed cultured paired hippocampal neurons to investigate the role of a splice variant of the α_2_δ-2 isoform in the trans-synaptic regulation of synapse formation and synaptic transmission, including short-term synaptic plasticity.

## Materials and reagents

**Animals**

Timed pregnant wild-type mice (BALB/c, gestational age 16–17 days; Charles River Laboratories, Sulzfeld, Germany).

**Biological materials**

Lentiviral particles, carrying RNA encoding for the α_2_δ-2_ΔE23 splice variant and soluble eGFP as fluorescent marker (Geisler et al., 2019). Lentiviral particles were generated as previously described (Nasri et al., 2014; Benskey and Manfredsson, 2016).

**Critical:** Lentiviruses are classified as a biosafety level 2 (BSL-2) organism.

**Materials**

1. Surgical scissors, sharp blunt, straight 14.5 cm (Fine Science Tools, catalog number: 14001-14)
2. Tissue forceps, slim 1 × 2 teeth 10 cm (Fine Science Tools, catalog number: 11023-10)
3. Fine scissors, sharp, curved 10.5 cm (Fine Science Tools, catalog number: 14061-10)
4. Fine scissors, sharp, straight 10.5 cm (Fine Science Tools, catalog number: 14060-10)
5. Dumont #5 standard forceps (Fine Science Tools, catalog number: 11251-30)
6. Dumont #5 biology forceps (Fine Science Tools, catalog number: 11252-30)
7. Vannas-Tübingen spring scissors (Fine Science Tools, catalog number: 15004-08)
8. 18 mm glass coverslips (Marienfeld Superior, catalog number: 0111580)
9. Rack for coverslips (custom build, Institute of Physiology, Medical University Innsbruck, Austria)
10. PTFE dish (Carl Roth, catalog number: K837.1)
11. 12.5 cm filter paper (Carl Roth, catalog number: AP86.1)
12. Hemacytometer (Neubauer, catalog number: Brand 717805)
13. 72 μm nylon mesh (Falcon, catalog number: 352350)
14. T75 flask (Falcon, catalog number: 353810)
15. Transfer pipette 3.5 mL (Sarstedt, catalog number: 86.1171.001)
16. 15 mL centrifuge tube (Falcon, catalog number: 352070)
17. 50 mL centrifuge tube (Falcon, catalog number: 352096)
18. 60 mm plastic Petri dish (Falcon, catalog number: 353802)
19. 60 mm Primaria plastic Petri dish (Falcon, catalog number: 353004)
20. 15 cm glass Petri dish (Duran, catalog number: 237555201)
21. Pasteur pipette (Assistent, catalog number: 40567002)
22. 5 mL serological pipette (Sarstedt, catalog number: 86.1253.001)
23. 10 mL serological pipette (Sarstedt, catalog number: 86.1254.001)
24. 25 mL serological pipette (Sarstedt, catalog number: 86.1685.001)
25. 1.5 mL miniature spray (Rene Lezard)
26. Borosilicate glass with filament (Sutter Instrument, model: BF150-75-10)
27. 2.5% trypsin (10×) (Gibco, catalog number: 15090-046)
28. 0.5% Trypsin-EDTA (10×) (Gibco, catalog number: 15400-054)
29. B-27 supplement (50×) (Gibco, catalog number: 17504-044)
30. GlutaMAX (Gibco, catalog number: 35050-038)
31. Horse serum (Gibco, catalog number: 16050-122)
32. PenStrep (Penicillin-Streptomycin) (Gibco, catalog number: 15140-122)
33. MEM (Gibco, catalog number: 41090-028)
34. Neurobasal medium (Gibco, catalog number: 21103-049)
35. HBSS (10×) (Gibco, catalog number: 14180-046)
36. HEPES 1 M solution (Gibco, catalog number: 15630-056)
37. Poly-L-lysine (Sigma, catalog number: P2636)
38. Ara-C (Sigma, catalog number: C6645)
39. DNase (Sigma, catalog number: DN-25)
40. Sodium pyruvate (Sigma, catalog number: P2256)
41. Paraffin (Carl Roth, catalog number: X880.1)
42. Gelatine (Fluka, catalog number: 48722)
43. Nitric acid (Carl Roth, catalog number: 4989.2)
44. Glucose (Carl Roth, catalog number: HN06.3)
45. Boric acid (Sigma, catalog number: B6768)
46. Borax (sodium tetraborate decahydrate) (Sigma, catalog number: B9876)
47. Sodium chloride (NaCl) (Carl Roth, catalog number: 3957.1)
48. Potassium chloride (KCl) (Carl Roth, catalog number: 6781.3)
49. Calcium chloride dihydrate (CaCl_2_·2H_2_O) (Carl Roth, catalog number: 5239.2)
50. Magnesium chloride hexahydrate (MgCl_2_·6H_2_O) (Sigma, catalog number: M0250)
51. Sodium hydroxide (NaOH) (Carl Roth, catalog number: 6771.3)
52. Potassium hydroxide (KOH) (Carl Roth, catalog number: 6751.1)
53. Gluconic acid, potassium salt (K-gluconate) (Carl Roth, catalog number: 4621.1)
54. HEPES (Carl Roth, catalog number: 6763.1)
55. EGTA (Sigma, catalog number: E3889)
56. ATP, magnesium salt (Sigma, catalog number: A9187)
57. GTP, sodium salt (Sigma, catalog number: G8877)

**Solutions**

1. Pyruvate solution, 100 mM, 50 mL (see Recipes)
2. 1% gelatine solution, 50 mL (see Recipes)
3. HBSS, 500 mL (see Recipes)
4. Glia medium, 500 mL (see Recipes)
5. Neuronal maintenance medium, 200 mL (see Recipes)
6. Neuronal plating medium, 200 mL (see Recipes)
7. 1% DNase solution, 100 mL (see Recipes)
8. Sodium borate buffer, 500 mL (see Recipes)
9. Poly-L-lysine solution, 1 mg/mL (see Recipes)
10. EGTA solution, 0.5 M, 1 mL (see Recipes)
11. Extracellular solution, 100 mL, adjust pH 7.4 with NaOH (see Recipes)
12. Intracellular solution, 20 mL, adjust to pH 7.2 with KOH (see Recipes)
13. Sodium hydroxide solution, 1 M, 5 mL (see Recipes)
14. Potassium hydroxide solution, 1 M, 5 mL (see Recipes)
15. 20% glucose solution, 50 mL (see Recipes)

## Recipes

1. **Pyruvate solution, 100 mM, 50 mL**
  **Table:** 

Reagent	Amount
Sodium pyruvate	550 mg
Milli-Q water	Add to the total volume of 50 mL
2. **1% gelatine solution, 50 mL**
  **Table:** 

Reagent	Amount
Gelatine	500 mg
Milli-Q water	Add to the total volume of 50 mL
3. **HBSS, 500 mL**
  **Table:** 

Reagent	Amount
HBSS (10×)	50 mL
PenStrep	5 mL
HEPES, 1 M	5 mL
Milli-Q water	440 mL
4. **Glia medium, 500 mL**
  **Table:** 

Reagent	Amount
MEM	430 mL
PenStrep	5 mL
Glucose, 20%	15 mL
Horse serum	50 mL
5. **Neuronal maintenance medium, 200 mL**
  **Table:** 

Reagent	Amount
Neurobasal medium	194 mL
B-27 supplement, (50×)	4 mL
GlutaMAX	2 mL
6. **Neuronal plating medium, 200 mL**
  **Table:** 

Reagent	Amount
MEM	172 mL
Pyruvate solution, 100 mM	2 mL
Glucose, 20%	6 mL
Horse serum	20 mL
7. **1% DNase solution, 100 mL**
  **Table:** 

Reagent	Amount
DNase	1 g
HBSS	100 mL
8. **Sodium borate buffer, 500 mL**
  **Table:** 

Reagent	Final concentration	Amount
Boric acid	50 mM	1.54 g
Borax (sodium tetraborate)	12.5 mM	2.376 g
Milli-Q water		Add to the total volume of 500 mL
9. **Poly-L-lysine solution, 1 mg/mL**
  **Table:** 

Reagent	Amount
Poly-L-lysine	100 mg
Sodium borate buffer	100 mL
10. **EGTA solution, 0.5 M, 1 mL**
  **Table:** 

Reagent	Amount
EGTA	190.2 mg
1 M KOH	Add to the total volume of 1 mL
11. **Extracellular solution, 100 mL, adjust pH 7.4 with 1 M NaOH solution**
  **Table:** 

Reagent	Final concentration	Amount
NaCl	137 mM	800.62 mg
KCl	3 mM	22.36 mg
Glucose	10 mM	180.2 mg
HEPES	10 mM	238.3 mg
CaCl_2_·2H_2_O	1.8 mM	26.46 mg
MgCl_2_·6H_2_O	2 mM	40.66 mg
12. **Intracellular solution, 20 mL, adjust to pH 7.2 with 1 M KOH solution**
  **Table:** 

Reagent	Final concentration	Amount
K-gluconate	125 mM	585.62 mg
KCl	10 mM	14.9 mg
HEPES	10 mM	47.66 mg
MgCl_2_·6H_2_O	1 mM	4.06 mg
EGTA solution, 0.5M	2 mM	80 μL
ATP (Mg)	4 mM	40.57 mg
GTP (Na)	0.3 mM	3.14 mg
13. **Sodium hydroxide solution, 1 M, 5 mL**
  **Table:** 

Reagent	Amount
NaOH	200 mg
Milli-Q water	Add to the total volume of 5 mL
14. **Potassium hydroxide solution, 1 M, 5 mL**
  **Table:** 

Reagent	Amount
KOH	280.6 mg
Milli-Q water	Add to the total volume of 5 mL
15. **20% glucose solution, 50 mL**
  **Table:** 

Reagent	Amount
Glucose	10 g
Milli-Q water	Add to the total volume of 50 mL

## Equipment

1. Stereo dissection microscope (Olympus, model: SZX2-ILLTQ)
2. Class II biological safety cabinet (Thermo Scientific, model: HERAsafe KS12)
3. Suction system (Welch, model: 112037-08)
4. Safety Bunsen burner (INTEGRA Biosciences, model: Fireboy Plus 144000)
5. Pipette controller (INTEGRA Biosciences, model: PIPETTEBOY acu 2)
6. Magnetic stirrer with heating (Phoenix Instrument, model: RMS-10HS)
7. CO_2_ incubator (Thermo Scientific, model: HERAcell 240i)
8. Water baths (VWR, catalog number: 462-0558)
9. pH meter (inoLab, model: pH 7110)
10. Micropipette puller (Sutter Instrument, model: P-97)
11. Microforge (Narishige, model: MF-830)
12. Ultrapure water purification system (Merck Millipore, model: Milli-Q^®^ EQ 7000)
13. Universal oven (Memmert, model: UF260)

**Experimental setup**

1. CleanBench lab table (TMC, model: TM-63-9012S)
2. Inverted fluorescent microscope (Olympus, model: IX-83, equipped with LUCPLFLN40XPH/0.6 objective, Lumencor LED lamp, eGFP filter)
3. Microscope dependent platforms (Sutter Instrument, model: MDM-83-2)
4. Micromanipulators (Sutter Instrument, model: MPC-325-2)
5. Two-channel patch clamp amplifier (HEKA Elektronik, model: EPC 10 USB Double)
6. Quick change chamber RC-41LP (Harvard Apparatus, catalog number: 64-0368)

## Software

1. PatchMaster v2x90.5 (HEKA Elektronik)
2. FitMaster v2x90.5 (HEKA Elektronik)

## Procedure

**Critical:** Steps A1e–A1k, A1m, A2d–A2f, A2h–A2l, A3b–A3h, and A3j–A3t should be performed under sterile conditions. Steps A2i–A2j should be performed in a biosafety level 2 cabinet.

- **Culturing paired hippocampal neurons in coculture with an astroglia feeder layer**
  Cell culture method was adapted from Kaech and Banker (2006) with slight modifications (Obermair et al., 2004; Geisler et al., 2019).
  1. Coverslip preparation
    **Critical:** It is necessary to start preparing the coverslips at least four days prior to the preparation of hippocampal cultures.
    - Place 18 mm coverslips in custom built racks and rinse in Milli-Q water.
    - Place racks with coverslips in concentrated nitric acid (63%) for 12 h.
    - Rinse racks with coverslips in Milli-Q water; perform two changes for 2 h each.
    - Transfer coverslips into 15 cm glass Petri dishes on top of 12.5 cm filter paper and sterilize with dry heat (200 °C for 10–12 h).
    - Transfer coverslips into 60 mm plastic Petri dishes (five per dish, see Figure 1).
*Note: To prevent coverslips from swimming off, put 10 μL of 1% sterile-filtered gelatine solution into the dish for each coverslip before transferring it into the dish. Coverslips can be easily transferred to the plastic dish by using a glass Pasteur pipette connected to the suction system.*
    - Melt sterile paraffin on the heated magnetic stirrer to 150 °C in a PTFE dish.
    - Apply three small drops of melted paraffin to each coverslip.
*Note: Paraffin can be transferred from PTFE dish to coverslips using fire-polished Pasteur pipettes (Figure 1). Dip the pipette into paraffin and touch the coverslip quickly before paraffin hardens. Repeat procedure for each paraffin dot.*
      **Figure 1.**
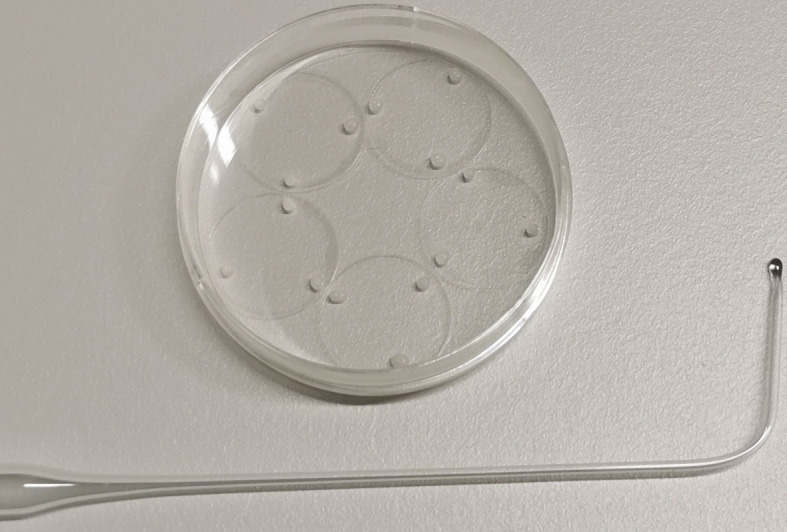
Fire-polished Pasteur pipette and 18 mm cover glasses with attached paraffin dots. Fire-polish the tip of the Pasteur pipette with a Bunsen burner. The tip should melt to form a glass bead with a diameter of approximately 2 mm (while heating the tip of the pipette, rotate it around the longitudinal axis to form a symmetrical bead), and the pipette should be bent by an angle of 50°–80° approximately 3 cm from the end of pipette.
    - Sterilize coverslips by UV irradiation for 30 min in the laminar flow (class II biological safety cabinet).
    - Spray poly-L-lysine solution using a 1.5 mL miniature spray bottle onto coverslips and let the poly-L-lysine dry at room temperature overnight.
      **Critical:** The poly-L-lysine solution should not be sprayed directly onto coverslips. Instead, small droplets of the solution, formed at the time of spraying, must settle down on the coverslips by gravity (Video 1). Dish with coverslips should be placed approximately 5 cm below the nozzle of the miniature spray.
      **Video 1.**
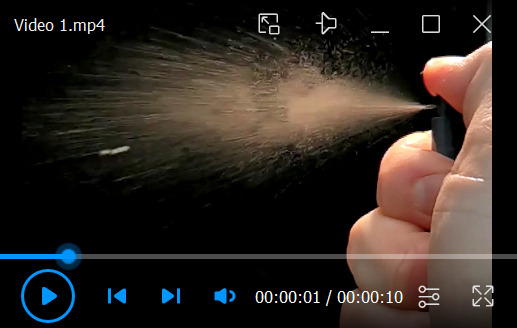
Small droplets of poly-L-lysine solution are formed during spraying (video at 32× reduced speed). The dish with the coverslips should be placed parallel to and beneath the main spray stream approximately 0.5–1 s after spraying.
    - Rinse dishes with poly-L-lysine-treated coverslips with sterile Milli-Q water; perform two changes for 2 h each.
    - Remove final rinse and add 4 mL of neuronal plating medium per dish.
    - Put dishes in cell culture incubator (37 °C, 5% CO_2_). The hippocampal neurons will be plated into these dishes.
    - Before plating neurons on coverslips, replace 4 mL of neuronal plating medium with fresh medium.
  2. Dissection and preparation of paired hippocampal cultures
    - Kill the pregnant mouse by cervical dislocation, remove uterus, and place it in a sterile 10 cm Petri dish. Remove fetuses from the uterus.
    - Quickly decapitate fetuses with scissors and place heads in HBSS (4 °C).
    - Under a dissecting microscope: dissect out brains, separate brain hemispheres, strip away the meninges, and cut out the hippocampi as shown in Video 2.
      **Video 2.**
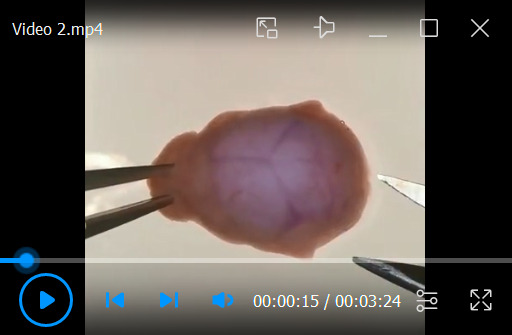
Dissection procedure of hippocampi from 16–17-day-old mouse embryos. This video was made in the MiMo Laboratory at the Karl Landsteiner University, which is a user facility in accordance with §16 TVG 2012, license number 2021-0.412.631, approved by the Austrian Federal Ministry of Science, Research and Economy.
*Note: For all steps following the dissection of hippocampi, all media and reagents should be prewarmed to 37 °C before use.*
    - Using the transfer pipette, place all the hippocampi from one litter in a 15 mL Falcon centrifuge tube. Bring the total volume to 4.5 mL with HBSS and add 0.5 mL of 2.5% trypsin (10×). Incubate in a water bath at 37 °C for 15 min.
    - Remove the trypsin solution, add 5 mL of HBSS (gently tap or swirl the tube to mix), and let stand for 5 min. Repeat this step twice, finally bringing the volume to 3 mL (4 mL if hippocampi from more than five brains are used).
    - Dissociate the cells by gently pipetting hippocampi up and down, first with a Pasteur pipette with a fire-polished tip to half the normal diameter, and then with a Pasteur pipette with a tip fire-polished to nearly a quarter the normal diameter. Continue pipetting gently until no chunks of tissue remain (approximately 7–8 times).
    - Determine the density of cells using a hemacytometer.
    - Add 25,000 cells to each of the dishes containing the poly-L-lysine-treated coverslips in neuronal plating medium (corresponding plating density is 880 cells/cm^2^).
    - For viral infection, add medium containing lentiviral particles to the dish with freshly added neurons.
*Note: To reach an approximate 50% viral infection efficiency, the volume and concentration of lentivirus added should be defined experimentally. Added medium with lentiviral particles should not exceed 1 mL per Petri dish containing 4 mL of neuronal plating medium.*
    - After 3 h and using forceps, transfer the coverslips with the neurons attached into dishes containing the glial cells in neuronal maintenance medium (see step A3). Turn the coverslips upside down so that the neurons are facing down, towards the glial cells.
    - To reduce glial proliferation, add Ara-C (5 μM) three days after plating the neurons.
    - Once a week, remove 2 mL of the neuronal maintenance medium and replace it with fresh medium.
    - For electrophysiological recordings, use cultured hippocampal neurons at the age 14–17 days in vitro (DIV) (Figure 2).
    **Figure 2.**
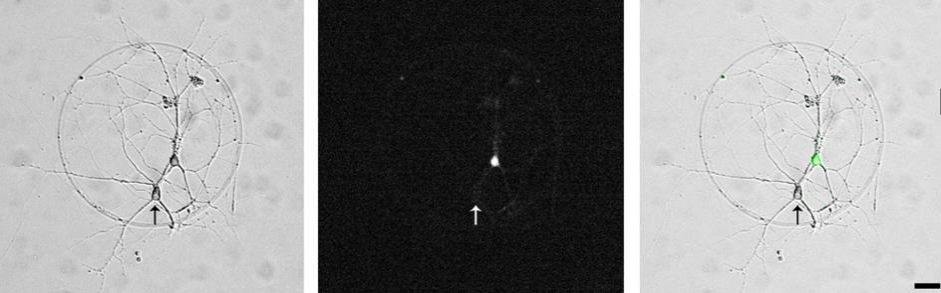
A pair of synaptically connected hippocampal neurons (left panel, phase contrast micrograph). One neuron is virally transfected with the α_2_δ-2 protein and soluble eGFP (middle, fluorescence micrograph and right panel, overlayed phase contrast and fluorescent micrographs), the other neuron is an untransfected control neuron (arrow). Scale bar, 20 μm.
  3. Preparation of astroglia feeder layer
    **Critical:** It is necessary to start preparing the glia feeder layer 14 days prior to the preparation of hippocampal cultures.
    - Prepare brain hemispheres as described in steps A2a–A2c.
*Notes:*
*i. If neuronal cultures are prepared on a regular basis, cells for the glia feeder layer can be prepared from the brains of mice used for the preparation of the neuronal culture.*
*ii. For all steps following the preparation of brain hemispheres, all media and reagents should be prewarmed to 37 °C before use.*
    - Mince brain hemispheres into small pieces with Vannas-Tübingen spring scissors.
    - Transfer minced tissue to a 50 mL Falcon centrifuge tube in a final volume of 12 mL of HBSS.
    - Add 1.5 mL of 1% DNase solution and incubate in water bath for 5 min at 37 °C.
    - Add 1.5 mL of 2.5% trypsin (10×) and incubate for 15 min at 37 °C. During trypsin treatment, dissociate tissue every 5 min with a 5 mL serological pipette (pipette tissue up and down 7–8 times).
      **Critical:** It is critical to add the DNase solution 5 min before adding the trypsin. Otherwise, DNase will be quickly degraded, resulting in abundant DNA material from minced brain tissues, which will strongly decrease the cell yield.
    - Add 3 mL of horse serum to inhibit trypsin activity.
    - Filter the combined supernatants through a 72 μm nylon mesh to remove any undissociated tissue.
    - Centrifuge supernatants at 200× *g* for 5 min at 4 °C and resuspend cells in 5 mL of glial medium.
    - Determine cell density with an hemacytometer.
    - Transfer 4,000,000 cells into a 75 cm^2^ T-flask (equivalent to approximately 1–1.5 brain hemispheres per flask). Add glial medium to a total volume of 13 mL. Put the flask in a cell culture incubator (37 °C, 5% CO_2_).
*Note: One 70%–80% confluent T-flask with astroglia cells will be enough to prepare 10 mm × 60 mm dishes of astroglia feeding layer.*
    - Three days after plating, replace glial medium in the flask completely with fresh medium.
    - Seven days after plating, shake the flask vigorously to dislodge microglia and remove them by washing (replace glia medium completely with fresh medium). Return the flask to the cell culture incubator.
      **Critical:** Slap the flask forcefully 2–3 times on a hard surface so that the medium foams up and the entire content appears messed up. Gently tapping the flask will not dislodge the microglia, which are typically accumulating on top of astrocytes.
    - When cells in the flask have reached confluence (usually 10 days after plating), remove the glial medium and wash with 10 mL of HBSS.
    - Remove HBSS, add 10 mL of 0.5% trypsin-EDTA (in HBSS), and put flask in cell culture incubator for 5 min.
    - Tap the flask gently on the side to detached remaining astrocytes, add 1 mL of horse serum, and place the flask upright to allow the cells to slide to the bottom.
    - Transfer glial cell suspension into a 50 mL Falcon centrifuge tube and centrifuge at 200× *g* for 5 min at room temperature.
    - Resuspend cell pellet in 40 mL of glial medium.
    - Add 4 mL of glial cell suspension per 60 mm Primaria plastic Petri dish. Put dishes in cell culture incubator (37 °C, 5% CO_2_).
    - On the next day, replace glia media in Petri dishes with fresh medium.
    - On the third day (one day before preparing hippocampal cultures), replace glia medium in dishes with astroglia layer with 6 mL of neuronal maintenance medium. Return dishes to the cell culture incubator.
- **Electrophysiological recordings of induced postsynaptic responses in paired cultured neurons**
*Notes:*
*i. The following steps require the experimenter to have knowledge and proficiency in two-channel patch clamp electrophysiology. All recordings and analyses were performed using PatchMaster software.*
*ii. To induce synaptic transmission within paired neurons, each neuron will be interchangeably stimulated with a depolarization using an action potential wave form, which needs to be recorded from wild-type (WT) neurons.*
  1. Recording of action potentials from WT hippocampal neurons
    - Pull glass patch pipette with 3–5 mm short taper and fire-polish its tip using a microforge. Resistance of the pipette should be 2–4 MΩ when filled with the intracellular solution.
    - Place coverslip with WT neurons into the low-profile chamber (Figure 3) and add 300 μL of extracellular solution.
      **Figure 3.**
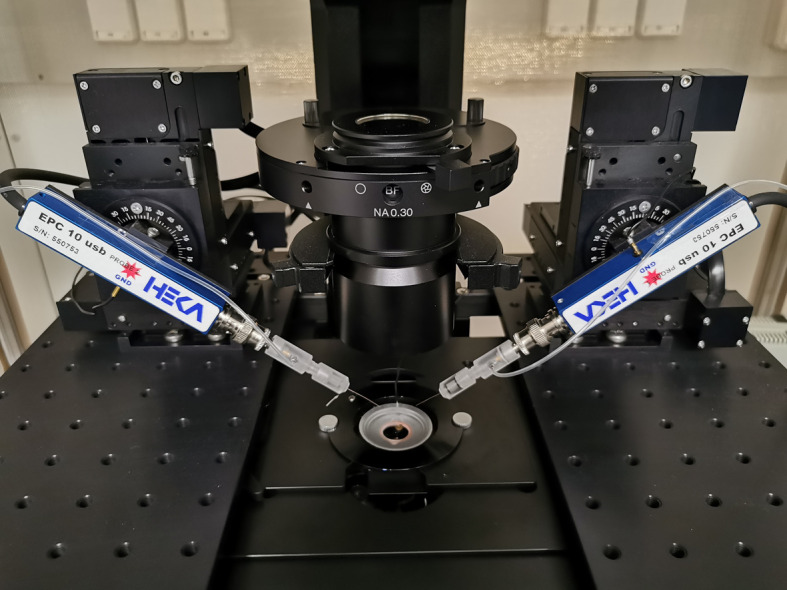
Experimental setup. Low-profile chamber is mounted on the precision controlled XY-stage. Probes 1 and 2 will be used for paired recordings.
    - Using probe 1, patch any neuron in voltage clamp mode using the whole-cell configuration. Holding potential (V_m_) should be set to -70 mV.
    - Switch from voltage clamp to current clamp mode.
    - Record spontaneous activity (generation of action potentials) of the neuron using the protocol shown in Figure 4.
*Note: If no spontaneous activity is observed in the neuron, depolarize neurons by continuous injection of an electrical current (5 pA, “I-membrane” box in “Amplifier” window). If necessary, increase amplitude of injected current with steps of 5 pA until the neurons start generating action potentials.*
      **Figure 4.**
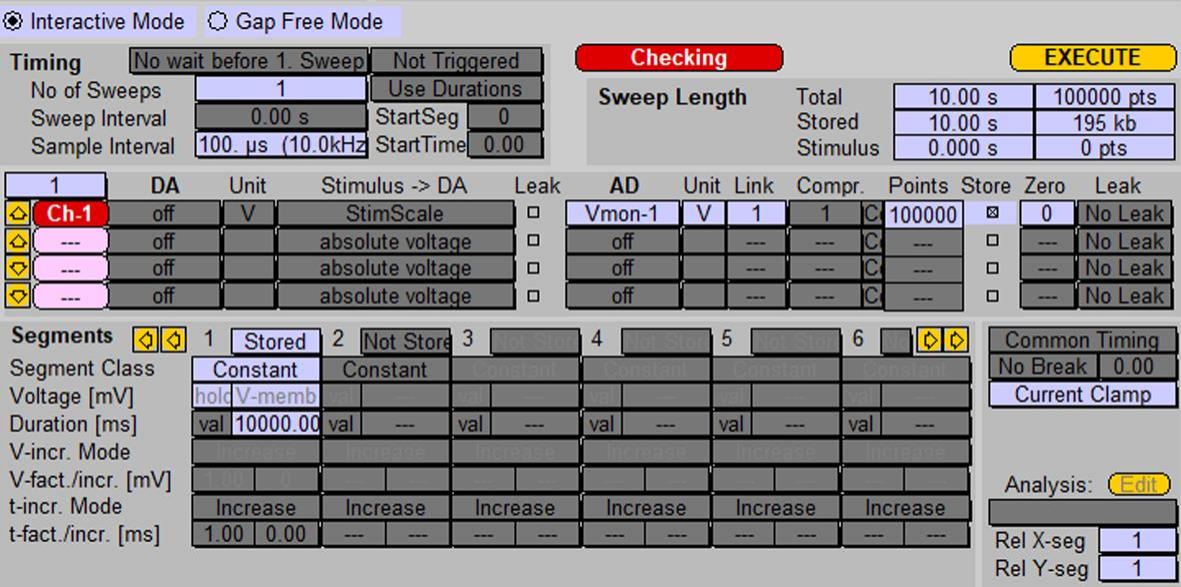
Protocol for recording spontaneous activity of neurons using probe 1. Screenshot of the “Pulse generator” window.
    - In the *Replay* window, choose the recorded trace and zoom in on one action potential.
    - Export the recorded action potential as a stimulation template file (Figure 5).
    **Figure 5.**
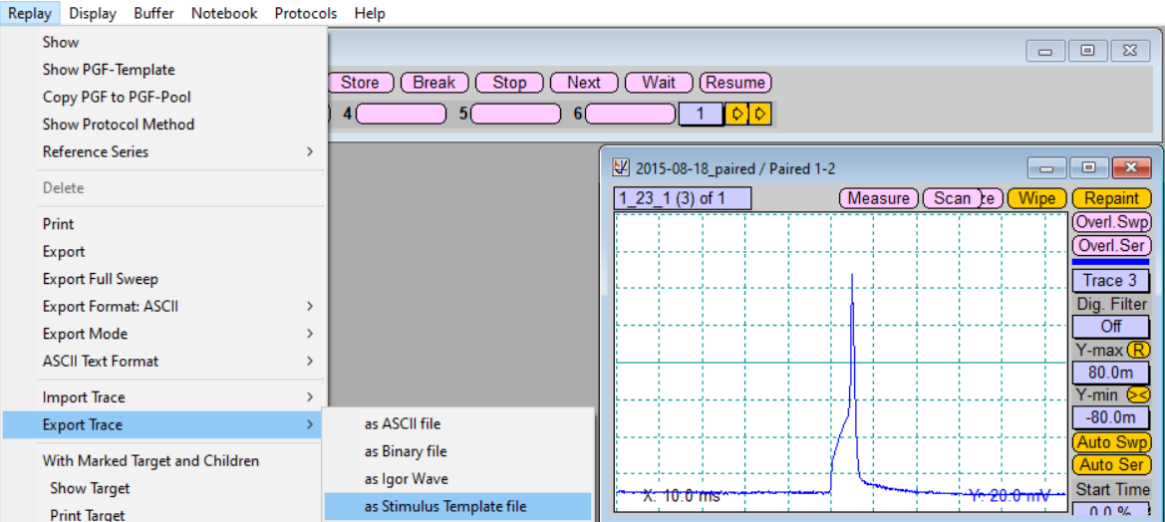
Export of the recorded action potential as stimulation template file
  2. Single stimulation of paired neurons
    - Prepare two stimulation protocols, as shown in Figure 6.
      *Note:* Duration [ms] *of recording time in* Segments *can be arbitrary but should be long enough to record a postsynaptic response.*
      **Critical:** If your stimulation protocol has the name “Stim,” then the corresponding stimulation template file must have the name “Stim_1.tpl” and it needs to be stored in the folder where the PatchMaster *.pgf file is located, for example at “C:\Program Files (x86)\HEKA\PatchMaster\.” Each stimulation protocol must have its own stimulation template file.
      **Figure 6.**
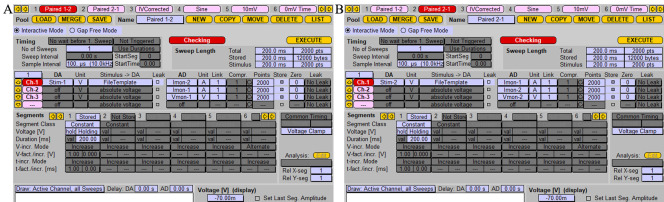
Protocol for recording induced synaptic response. A. Stimulation protocol for stimulating the cell patched with probe 1 and postsynaptic response recording from cell patched with probe 2. “Stim-1” should be chosen as a stimulus in the “DA” section; in the “AD” section, stimulation signal and postsynaptic responses are recorded as Vmon-1 and Imon-2, respectively. B. Stimulation protocol for stimulating the cell patched with probe 2 and postsynaptic response recording from the cell patched with probe 1. Screenshot of the “Pulse generator” window. “Stim-2” should be chosen in the “DA” section; Vmon-2 and Imon-1 should be chosen in the “AD” for recording.
    - Pull glass patch pipettes with 3–5 mm short taper and fire-polish its tip using a microforge. Resistances of pipettes should be 2–4 MΩ when filled with the intracellular solution.
    - Place coverslip with neurons into the chamber and add 300 μL of extracellular solution.
    - Patch both neurons in the voltage clamp mode using whole-cell configuration. Holding potential (V_m_) should be set to -70 mV for both cells.
    - Alternately, apply the stimulation protocols for both patched cells. Induced postsynaptic response will be recorded from the respective unstimulated cell (Figure 7).
    **Figure 7.**
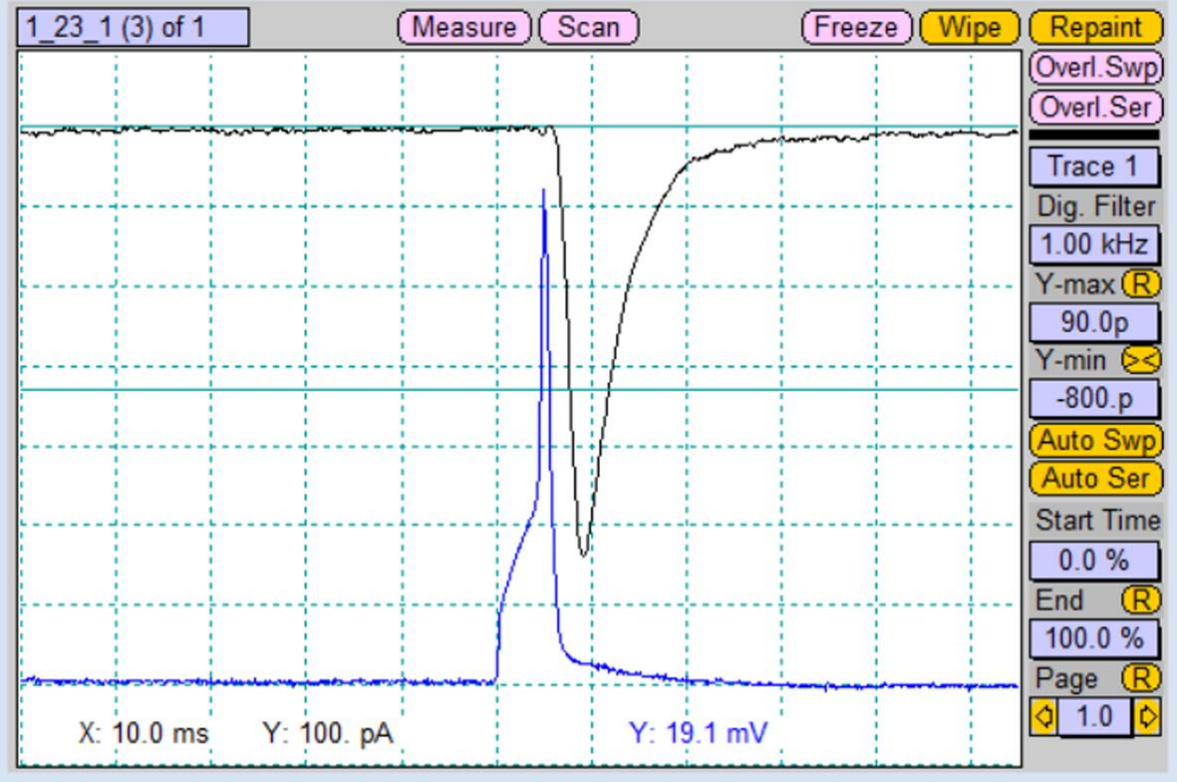
Recorded postsynaptic current (black trace) from the paired postsynaptic neuron after stimulation of the paired presynaptic neuron with an action potential (blue trace). Screenshot of “Oscilloscope” window.
  3. Paired-pulse stimulation of paired neurons
    - Prepare protocol for paired-pulse stimulation, as shown in Figure 8.
      **Figure 8.**
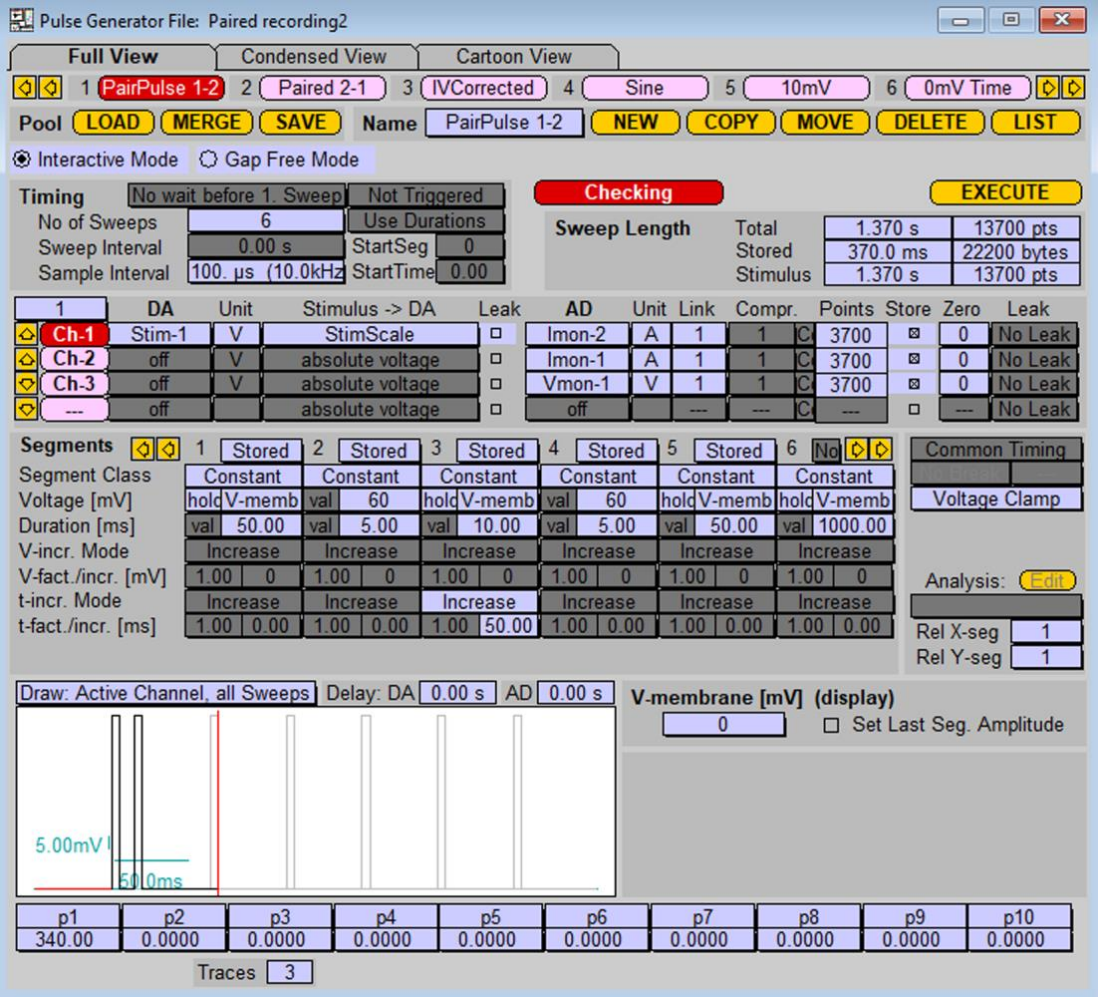
Stimulation protocol for paired-pulse stimulation of the cell patched with probe 1 and postsynaptic response recording from the cell patched with probe 2. Stimulation protocol represents a series of stimulations by two sequential depolarizations from -70 mV to 60 mV (5 ms duration each) with increasing inter-pulse interval. To stimulate cells on probe 2 with the response recording on probe 1, replace values in “DA” and “AD” sections (1 into 2, 2 into 1).
    - After application of a single stimulation alternately, apply the pair-pulse stimulation protocol to both presynaptic/postsynaptic cell configurations.

## Data analysis

1. To analyze amplitudes of recorded induced postsynaptic responses, create the two functions “Minimum” and “Series time” in the window *Analysis*, as shown in Figure 9.
*Note: The function “Minimum” is used to analyze postsynaptic currents in response to excitatory (glutamatergic) synaptic transmission. To analyze inhibitory (GABAergic) synaptic transmission, use the function “Maximum,” as the postsynaptic currents have opposite polarity of the peak current.*
  **Figure 9.**
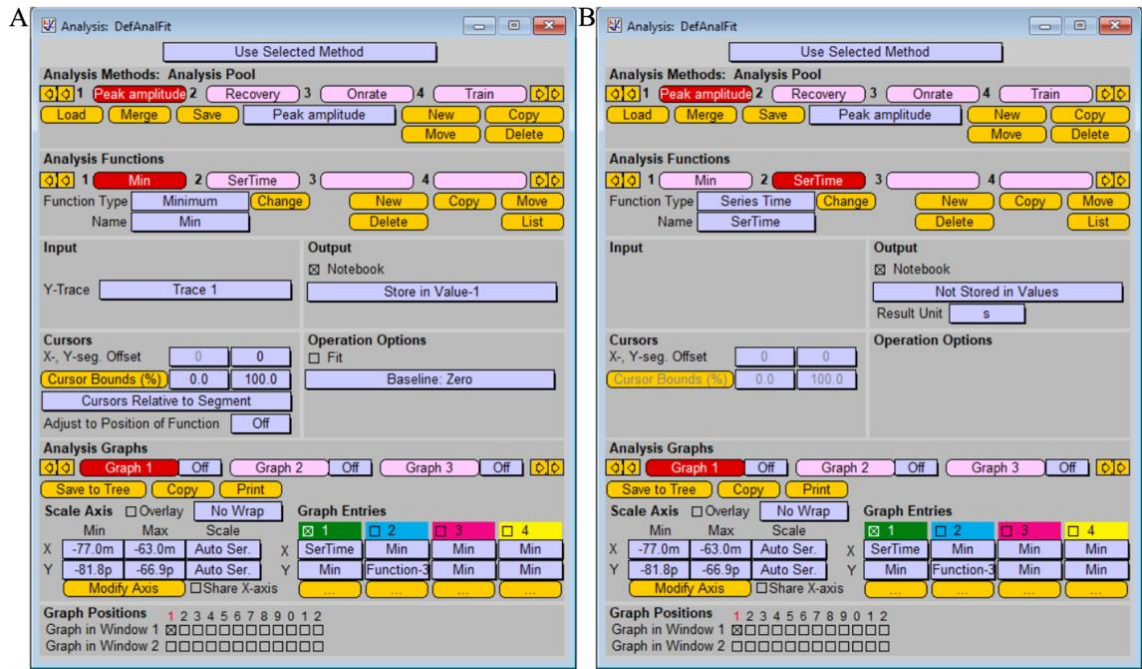
Analysis protocol for measuring current amplitude of postsynaptic response functions “Minimum” (A) and “Series time” (B). Screenshot of the “Analysis” window.
2. After application of the single stimulation protocol, read peak value of the synaptic current in *Notebook* window.
3. For the analysis of individual peak amplitudes after pair-pulse stimulation, chose the segment of the recorded trace to be analyzed (change *Cursor Bounds (%)* in *Analysis* window) and replay trace for analysis of each peak (Figure 10).
*Note: Alternatively, analysis of the amplitude of recorded postsynaptic response can be performed using the FitMaster software.*
  **Figure 10.**
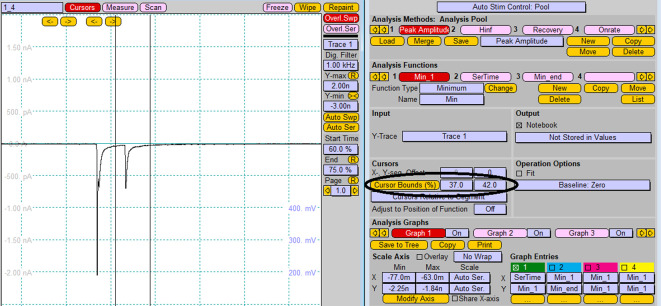
Analysis of individual peak amplitudes after pair-pulse stimulation. Changing of cursor bounds is represented by the vertical lines on the *Oscilloscope* window.

## General notes and troubleshooting

**Figure 11.**
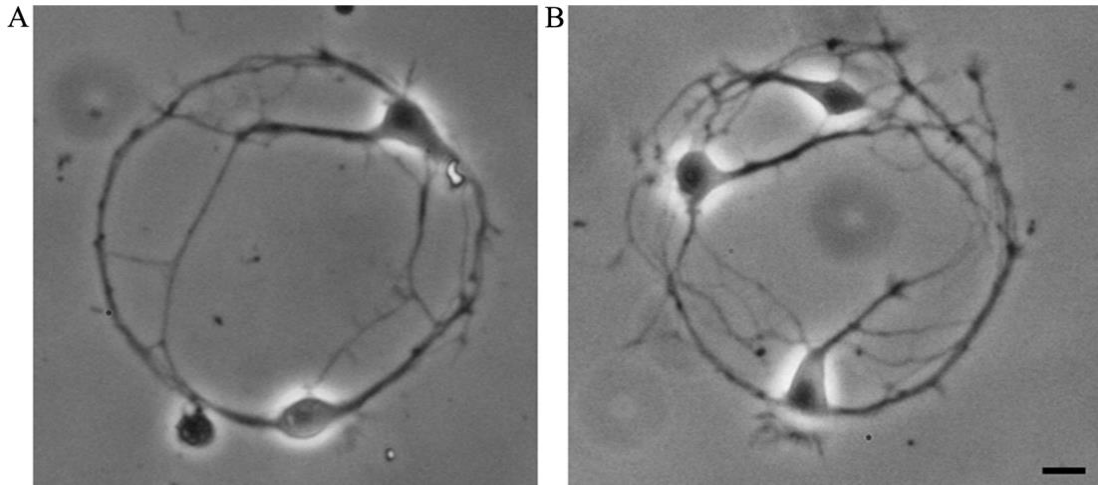
Neuronal networks of cultured hippocampal neurons, formed by two (A, aim of the protocol) or three (B, plating density should be reduced) cells. Scale bar, 10 μm.

While performing experiments according to the current protocol, the experimenter may experience two types of problems related to the formation of neuronal pairs and the efficiency of viral infection. Both problems can be easily solved. If more than two neurons form networks on most of the poly-L-lysine spots (Figure 11), the plating density should be reduced (see step A2h). Increasing plating density is required if the majority of poly-L-lysine spots contain only one neuron.

To successfully perform experiments on paired neurons in which one neuron was genetically modified, the efficiency of viral infection should be approximately 50%. If both neurons are virally infected or none are infected, it is necessary to decrease or increase, respectively, the concentration of the virus that is added to the freshly plated hippocampal neurons (step A2i).

## Validation of protocol

The presented protocol was developed and successfully employed for the research work published in Geisler et al. (2019), in order to analyze the consequences of altered synaptic wiring induced by the α_2_δ2_ΔE23 isoform (section “Reduced synaptic transmission in aberrantly wired synapses,” Figure 12 in the article).
